# The American Medical Society of Paris

**Published:** 1854-10

**Authors:** 


*The American Medical Society of Paris.*—We have hitherto neglected to notice the communication which this body sent to the American Medical Association at its St. Louis meeting. Whatever may have been the motives of the memorial, and in however honest a spirit it may have been conceived, it was certainly in very bad taste, and ill-calculated to effect any good purpose. Whatever may be the opinion of the young gentlemen who make up the Parisian society, it is by no means sure that the general standard of medical attainment is lower in this country than in France, or other parts of Europe. It is not probable that the earnest and practical American mind should content itself with a lower grade of such acquirement as is practically useful, than that of our trans-Atlantic brethren.

One great mistake is made in this connection. The profession of Paris is not the profession of France. The study of specialties in that city has reached a perfection which may well dazzle the eye unaccustomed to the concentration of the whole medical talent of a country in a single town. In America talent is diffused; but, relatively to the population, we believe that there is more medical skill here than in France.

Count up the great names of France, and then enumerate the men of America who equal them, and though a disparity may be found in the list of celebrities, yet, were the rank and file of the two nations to be placed side by side we should have no need to blush.

Neither have we any reason to disparage the teachers and specialists of our country. Physical diagnosis is better understood here than in Paris; and we not unfrequently hear of blunders in diagnosis in the hospitals of that city, which require all the eclat and reputation of their perpetrators to sustain them. We could enumerate many American names which will compare favorably with those of the brightest of European celebrities.

But it was not to indulge in our national sin of boasting, that we commenced this article. It was to offer a few thoughts, suggested and confirmed by an editorial paper in the American Medical Monthly on the vexed subject of medical education. The letter of the American Medical Society of

Paris recommended the appointment of a board of examiners by the state societies, who should alone have the power of conferring degrees; thus depriving the colleges of that function.

Two or three queries arise. Who is to pay this board of examiners? Will they possess any better means of ascertaining the qualifications of candidates than the present system offers? Will they be more faithful in the discharge of their duties?

The first question is sufficient to destroy the plan of the Paris society. In the states of Pennsylvania and New York, the number of students to be examined count by hundreds. Many weeks or months would be required to do justice to this inquiry. These students come before the board unknown, and without any possibility of forming an opinion as to their qualifications, except by a series of questions and answers which a little preliminary cramming would enable any man to pass. In the colleges the student has been for months in daily association with his examiner. He has been upon the quiz list, and his general character, calibre, and conduct, are known. He is rated fairly. If he is a well-stuffed blockhead, prepared to answer the usual questions with parrot-like accuracy, the acumen of his teacher discovers the fact, and varies his examination accordingly. Or if he is, one of that modest, diffident sort, who are frightened out of all capacity, due allowance is made. Nowhere can an examination be conducted so rigidly, and yet so fairly, as by the teacher who is familiar with the candidate. There is nothing in a system of state-examiners to compensate for the loss of these advantages, unless it be that the graduation fee acts as a bribe to the colleges. Those who have any faith in this stale charge are entirely welcome to their opinion.

There is not a little misconception as to the comparative excellence of the medical profession. It is demanded and expected that none but qualified men should enter it. This is assuredly desirable, and every precaution should be taken to secure its attainment. But somehow or other unqualified men do get into all occupations. "One-horse-lawyers" are proverbially frequent, and yet they have all passed a board of state examiners. Clergymen who have most egregiously mistaken their mission are equally common; and so on through every profession, trade, or business, incompetent men come in for a share in the honors and profits. When human nature shall have become perfect, when humility and diffidence are stronger motives than hope and ambition, this will be bettered. In the meantime, it is, perhaps, as well to wait and content ourselves with the performance of our individual duties.

We have all along spoken of the colleges as responsible for the character character of their alumni. But is it not possible that some blame may rest upon other parties? We have now in our mind two students. One is faithful and painstaking. No advantage within his means has been neglected. The dissecting-room, the *clinique*, the lecture room, and the midnight lamp have year after year been the silent witnesses of his determination to succeed. And after all this he will pass an examination so poorly, that a board of examiners, who were strangers to him, would be in duty bound to reject him. And yet we have, through long acquaintance, a strong assurance that he will make a competent and useful practitioner. We know another, whose studies have been the occupation of dull days when no amusement offered, and who has run rapidly through with the abstruse and fanciful, to the neglect of the plain and practical, who will, in spite of all this, pass a most creditable examination.

Give us some test for industry. Let us have some scale by which we may weigh true acquirement properly, and be able to prophesy as to whether or no the attainment we behold is solid learning, the fruit of studious habits which will continue through a life of practice, and fewer poor students would pass. H.

